# Leigh syndrome caused by mutations in *MTFMT* is associated with a better prognosis

**DOI:** 10.1002/acn3.725

**Published:** 2019-02-17

**Authors:** Hannah Hayhurst, Irenaeus F. M. de Coo, Dorota Piekutowska‐Abramczuk, Charlotte L. Alston, Sunil Sharma, Kyle Thompson, Rocio Rius, Langping He, Sila Hopton, Rafal Ploski, Elzbieta Ciara, Nicole J. Lake, Alison G. Compton, Martin B. Delatycki, Aad Verrips, Penelope E. Bonnen, Simon A. Jones, Andrew A. Morris, David Shakespeare, John Christodoulou, Dorota Wesol‐Kucharska, Dariusz Rokicki, Hubert J. M. Smeets, Ewa Pronicka, David R. Thorburn, Grainne S. Gorman, Robert McFarland, Robert W. Taylor, Yi Shiau Ng

**Affiliations:** ^1^ Wellcome Centre for Mitochondrial Research Institute of Neuroscience Newcastle University Newcastle upon Tyne United Kingdom; ^2^ Department of Neurology Erasmus Medical Centre Rotterdam Netherlands; ^3^ Department of Paediatrics, Nutrition and Metabolic Diseases The Children's Memorial Health Institute Warsaw Poland; ^4^ Department of Medical Genetics The Children's Memorial Health Institute Warsaw 04‐730 Poland; ^5^ Victorian Clinical Genetics Service and Murdoch Children's Research Institute Parkville Victoria 3052 Australia; ^6^ Department of Paediatrics University of Melbourne Melbourne Victoria 3052 Australia; ^7^ Department of Neurology Canisius Wilhelmina Hospital Nijmegen The Netherlands; ^8^ Department of Molecular and Human Genetics Baylor College of Medicine Houston Texas; ^9^ Manchester Centre for Genomic Medicine Manchester University NHS Foundation Trust Manchester Academic Health Sciences Centre Manchester UK; ^10^ Neuro‐Rehabilitation Unit Royal Preston Hospital Preston United Kingdom; ^11^ Department of Clinical Genomics Research Schools GROW and MHeNS Maastricht University Maastricht The Netherlands

## Abstract

**Objectives:**

Mitochondrial methionyl‐tRNA formyltransferase (MTFMT) is required for the initiation of translation and elongation of mitochondrial protein synthesis**.** Pathogenic variants in *MTFMT* have been associated with Leigh syndrome (LS) and mitochondrial multiple respiratory chain deficiencies. We sought to elucidate the spectrum of clinical, neuroradiological and molecular genetic findings of patients with bi‐allelic pathogenic variants in *MTFMT*.

**Methods:**

Retrospective cohort study combining new cases and previously published cases.

**Results:**

Thirty‐eight patients with pathogenic variants in *MTFMT* were identified, including eight new cases. The median age of presentation was 14 months (range: birth to 17 years, interquartile range [IQR] 4.5 years), with developmental delay and motor symptoms being the most frequent initial manifestation. Twenty‐nine percent of the patients survived into adulthood. MRI headings in *MTFMT* pathogenic variants included symmetrical basal ganglia changes (62%), periventricular and subcortical white matter abnormalities (55%), and brainstem lesions (48%). Isolated complex I and combined respiratory chain deficiencies were identified in 31% and 59% of the cases, respectively. Reduction of the mitochondrial complex I and complex IV subunits was identified in the fibroblasts (13/13). Sixteen pathogenic variants were identified, of which c.626C>T was the most common. Seventy‐four percent of the patients were alive at their last clinical review (median 6.8 years, range: 14 months to 31 years, IQR 14.5 years).

**Interpretation:**

Patients that harbour pathogenic variants in *MTFMT* have a milder clinical phenotype and disease progression compared to LS caused by other nuclear defects. Fibroblasts may preclude the need for muscle biopsy, to prove causality of any novel variant.

## Introduction

Leigh syndrome (LS) is one of the most common paediatric mitochondrial disorders, with a prevalence of 1:32,000–40,000.^1^ It is a progressive neurodegenerative disorder commonly characterized by the onset of symptoms such as hypotonia, spasticity or developmental delay between the ages of 3 and 12 months, with 83% of the patients presenting before the age of 2 years.^2^, ^3^ To date, pathogenic variants in more than 80 genes have been shown to cause LS although these only account for approximately one half of all cases of the disease.^4^, ^5^ The survival of patients with LS is typically poor, with a median survival of 2.4 years.^3^

In 2011, Tucker and co‐workers identified three patients from two unrelated families with segregating, compound heterozygous variants in *MTFMT* who presented with LS.^6^ The mitochondrial methionyl‐tRNA formyltransferase (*MTFMT*) gene encodes the mitochondrial protein of the same name, which is required for the initiation of translation of mtDNA‐encoded proteins. MTFMT is responsible for formylating Met‐tRNA^Met^; in mammalian mitochondria, there is only a single Met‐tRNA, so the ratio of fMet‐tRNA^Met^ (for initiation) to Met‐tRNA^Met^ (for translation elongation) must be tightly controlled.^7^ MTFMT deficiency can therefore cause impaired mitochondrial protein synthesis resulting in multiple respiratory chain deficiencies. Further cases have since been described, including a report of the phenotypic spectrum of 11 patients with proven pathogenic variants in *MTFMT*.^8^

In this study, we report eight new cases with bi‐allelic *MTFMT* variants. By combining our patients with other previously reported cases, we sought to elucidate the full spectrum of clinical, radiological and molecular genetics findings, and evaluate the survival status of individuals with *MTFMT*‐related mitochondrial disease.

## Methods

### Subjects

New patients and additional data from previously published cases were identified from the clinical and diagnostic centers with expertise in mitochondrial disease across Europe (UK, Poland, and Netherlands), Australia and USA. Clinical, radiological and molecular genetic data were captured using a standardized pro forma. This study was performed in accordance with the World Medical Association's Declaration of Helsinki and research and ethical guidelines issued by each institution. A systematic review of the available literature was conducted to ascertain previously published cases. Authors of previously published cases were contacted to provide additional clinical and neuroimaging data (that were missing).

### Histochemistry, quadruple immunohistochemistry and immunoblotting

Tissue obtained from muscle biopsies was subjected to histochemical analysis through the use of cytochrome *c* oxidase (COX), succinate dehydrogenase (SDH) and sequential COX/SDH reactions to allow analysis of COX‐deficient fibers as a marker of mitochondrial respiratory chain deficiency (P1‐4).^9^ For patients six to nine, histoenzymatic methods for assessment of the activities of individual mitochondrial respiratory chain complexes in muscle were employed as previously described.^10^ Quadruple immunofluorescent histochemical analysis, as described elsewhere,^11^ was performed on a muscle biopsy (P3). Western blotting was performed in one patient (P5) using published protocols.^6^

### Biochemical analysis

Mitochondrial respiratory chain complex activities were determined spectrophotometrically using patient skeletal muscle homogenates (P1‐4; P6‐9; P11‐14) and fibroblasts (P5) as previously described.^12^, ^13^, ^14^

### Identification of pathogenic *MTFMT* variants

The majority of the patients were diagnosed via exome sequencing or a custom MitoExome panel.^6^, ^15^, ^16^, ^17^ Two patients were diagnosed using a custom Ampliseq panel and Ion Torrent PGM sequencing and direct sequencing of *MTFMT*, respectively. All *MTFMT* variants detected by NGS methodologies were confirmed using Sanger sequencing; variant nomenclature is according to GenBank Accession Number NM_139242.3.

### Statistical analysis

To determine putative predictors of survival, Kaplan‐Meier analysis and Cox‐regression analysis were applied. All analyses were performed using SPSS software (V 22.0) and Minitab (V 17). The significance level was determined at ≤ 0.05 level.

## Results

### Patients

Thirty‐eight patients with known variants in *MTFMT* were identified: eight new cases (P1,2,5,6,7,8,10,11), nine previously published cases with additional clinical information (P3,4,9,12,13,14,15,16,17), and a further 21 patients were identified through the literature search (Table S1). Clinical data were available for further analysis in 34 patients (Table 1). A clinical synopsis for 10 patients is available in Data S1.

**Table 1:** Summary of clinical features (*n* = 34)

	Previously reported	New cases	Total
No of individuals; pedigrees	26; 23	8; 8	34; 31
Prenatal/Antenatal			
Premature	5	1	6
SGA	4	3	7
Pathological signs at birth	4	3	7
Microcephaly	7	3	10
Hypospadias	0	3	3
Developmental delay/regression	20	7	27
Seizures	3	3	6
Gait abnormality	14	5	19
Hypotonia	11	6	17
Abnormal reflexes	12	4	16
Dystonia	5	3	8
Tremor^a^	4	0	4
Ocular features	14	6	20
Feeding difficulties	4	6	10
Respiratory problems	4	6	10
Cardiac dysfunction	12	5	17
Lactic acidosis	19	8	27
Acute exacerbations	15	5	20
ITU admission	6	4	10

SGA, small for gestational age. Ocular features included nystagmus, strabismus, decreased visual acuity, ophthalmoplegia and gaze palsies. Respiratory problems included apnoea, hypoventilation and hyperventilation.

a One patient (P14 in Table S1) exhibited the triad of Parkinsonism.

### Disease onset and presenting symptoms

The median age of onset was 14 months (range: birth to 17 years, interquartile range: 4.5 years). Twenty patients (59%) had developmental delay or regression at presentation: global psychomotor delay involving motor movement and speech (*n* = 12), motor delay (*n* = 5), speech delay (*n* = 2) and developmental regression only (*n* = 1). Two patients presented with autism spectrum disorder. Sixteen patients (47%) presented with motor symptoms, including motor delay, gait instability, hypotonia and weakness. A third of the patients presented with ocular symptoms including nystagmus, strabismus and optic atrophy. Ten patients (29%) had feeding difficulties.

### Clinical features throughout disease course

Ninety‐four percent of (32 of 34) the patients demonstrated abnormal motor findings throughout their disease course (Table**** 1). Two patients had features of Parkinsonism including tremor, bradykinesia, rigidity, paucity of facial expressions and poverty of gait (Patients 14 and 25).^8^, ^18^ Axonal, sensory neuropathy was identified in one patient. The assessment of mobility was available for 15 patients; nine patients (60%) were still ambulatory (unaided) during their clinic assessment (mean age 17 ± 9 years, range 6–31 years).

Ocular symptoms were present in 20 patients (59%): strabismus including subacute, unilateral gaze palsy (*n* = 10), nystagmus (*n* = 7), optic atrophy (*n* = 4), reduced visual acuity (*n* = 4), ptosis (*n* = 2), pigmentary degeneration of retina (*n* = 1) and hemianopia (*n* = 1). Six patients (17%) had epilepsy; these included focal seizures, generalized tonic–clonic seizures and convulsive status epilepticus.

Twenty‐four out of 29 (83%) had elevated serum lactate (83%) (range 2.7 to 14.3 mmol/L, normal < 2.2 mmol/L). For the 24 patients with reported CSF lactate levels, 23 patients (96%) had elevated levels (range 3.3–7.8 mmol/L, normal < 2.2 mmol/L). Eighteen of 34 patients (53%) had documented abnormal cardiac findings. Structural cardiac abnormalities were identified in 12 patients: hypertrophic cardiomyopathy (*n* = 7), ventricular septal defects (VSD) (*n* = 3), noncompaction cardiomyopathy (*n* = 1), pulmonary stenosis (*n* = 1) and aortic regurgitation (*n* = 1). One patient had both aortic regurgitation and a VSD. Abnormal cardiac rhythm and conduction abnormalities were reported in five patients: Wolff–Parkinson–White syndrome (*n* = 3), symptomatic sinus tachycardia (*n* = 1), and supraventricular tachycardia (*n* = 1). Two patients developed bradycardia concomitantly with the apnoeic episodes, likely secondary to the subacute brainstem dysfunction related to LS.

### Acute decompensation requiring hospital admission

**Figure 1:**
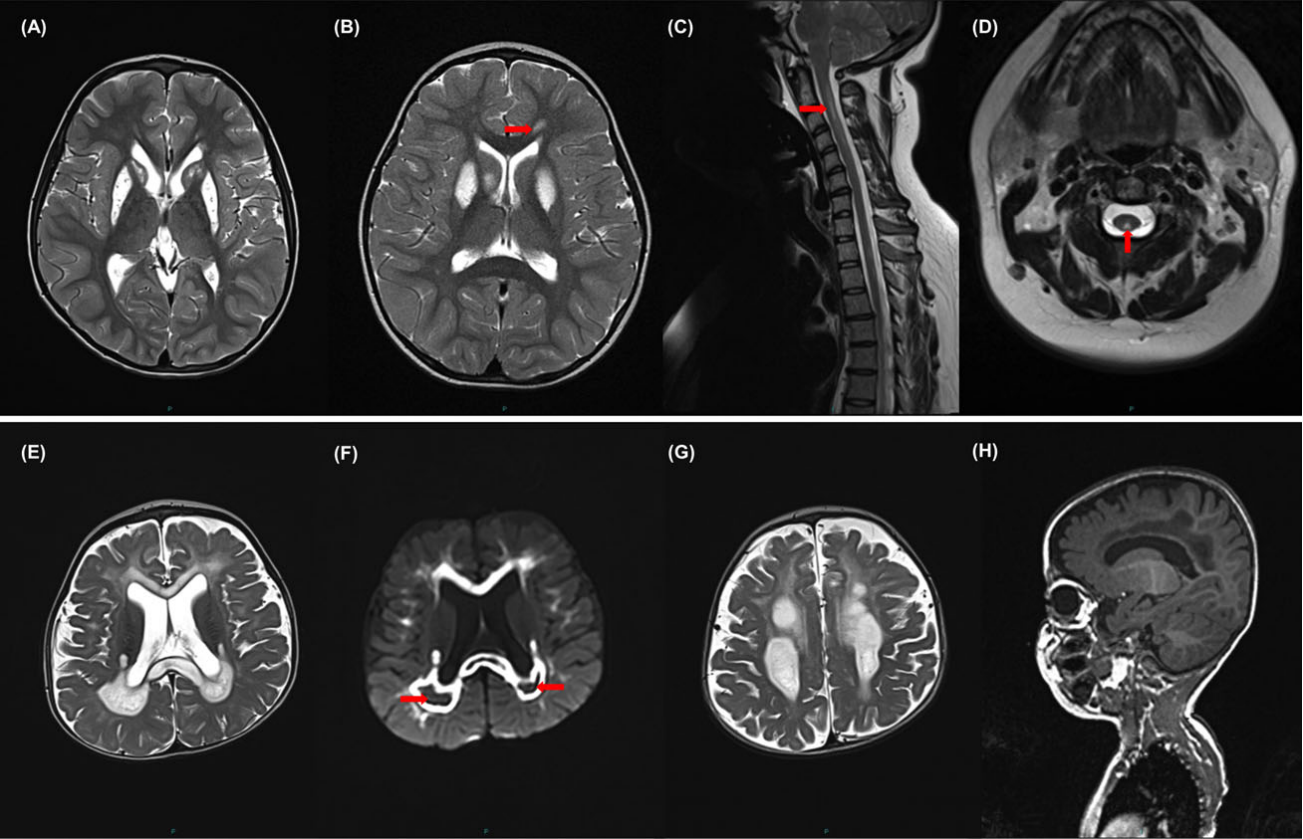
MRI head and cervical cord. T2‐weighted axial views show symmetrical hyperintensities in the striatum (A) (P4), hyperintensities in bilateral putamen, right caudate nucleus and a small white matter change in the left genu of corpus callosum (arrow) (B) (P2). T2‐weighted sagittal view shows an intrinsic T2 hyperintensity spanning C1‐4 level (C), with a corresponding change identified on the axial view (D) (P3). Extensive T2‐hyperintensities with areas of cavitation seen in the genu and splenium of the corpus callosum (E) and the posterior periventricular white matter with restricted diffusion, but not within the areas of cavitation (arrows) (F). (G) Large cystic changes with surrounding hyperintense T2 signal abnormality are present in the deep white matter. T1‐weighted sagittal view shows the confluent signal abnormality of the corpus callosum (H) (P1).

Fifty‐nine percent of patients (20 of 34) had acute exacerbations/decompensation requiring hospital admission throughout their disease course, and ten patients who had acute exacerbations required admission to an intensive care unit (ICU) setting. Fifty‐five percent of decompensations (*n* = 11) were due to respiratory complications. Infections accounted for acute hospital admissions in ten patients, gait disturbance in six, seizures in five and poor nutrition/dehydration in three patients. One patient manifested with subacute onset of arm and chest pain, with MRI of whole spine showing intrinsic cord signal abnormalities (Fig.**** 1) that partially resolved clinically and radiologically with supportive treatment only.

### Neuroimaging

Neuroimaging data were available for 33 patients (Table 2 and Fig. 1). In the 19 patients with white matter changes, 12 also had changes in the basal ganglia. Among the five patients who had spinal imaging, three had intrinsic cervical and/or thoracic spinal cord signal changes. In one patient, the rarefaction of white matter was accompanied by cavitating lesions with peripheral enhancement in the centrum semiovale (Fig.**** 1).

**Table 2:** Summary of cranial MRI findings (*n* = 33)

Imaging findings (*n* = 33)	Frequency (%)
Caudate	12 (36)
Putamen	18 (55)
Globus pallidus	10 (30)
Any part of basal ganglia	21 (64)
Midbrain	14 (42)
Pons	4 (12)
Medulla	5 (15)
Any part of brainstem	16 (48)
Basal ganglia and brainstem changes	10 (30)
Corpus callosum	10 (30)
White matter changes	18 (55)
Patchy, non‐specific	14 (42)
Leukodystrophy	4 (12)
Crus cerebri	4 (12)
Spinal cord (*n* = 5)	3 (60)

Note only five patients had imaging of their spinal cord performed or commented.

Six patients (18%) had neither basal ganglia nor brainstem abnormalities even though their clinical presentation and disease trajectory was consistent with LS (P1, P19, P24, P28, P31 and P37). Four of these patients had extensive white matter abnormalities identified on MRI head (P1, P24, P28 and P31), one had a normal MRI head and MR spectroscopy at the age of 4 years (P37), and one patient had multiple ischaemic stroke changes with neurovascular imaging showing changes suggestive of Moya‐moya disease (P19).^19^ The median age of patients with either basal ganglia or brainstem abnormalities was significantly older than those without any changes (16.5 vs. 5 years, *P* = 0.04).

### Evaluation of respiratory chain complexes in muscle biopsy and fibroblasts

**Figure 2:**
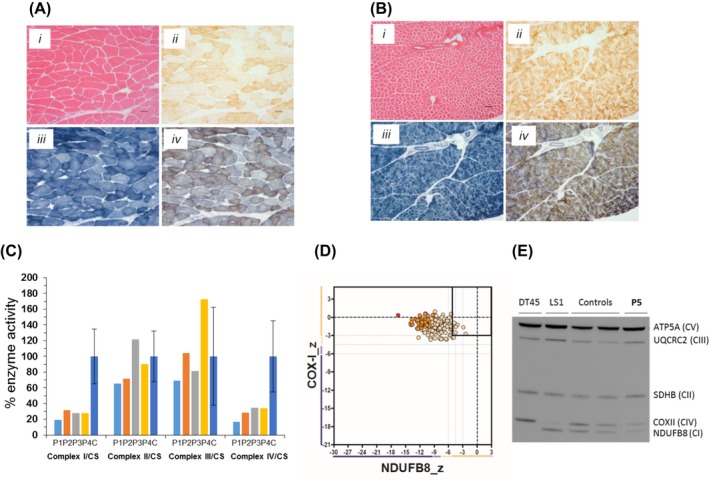
Histopathological and biochemical analyses of patient muscle biopsies. (A and B) Histopathological analysis of patient skeletal muscle sections showing hematoxylin and eosin (H&E) staining (*i*), cytochrome *c* oxidase (COX) histochemistry (*ii*), succinate dehydrogenase (SDH) histochemistry (*iii*) and sequential COX‐SDH histochemistry (*iv*) for P3 (A) and P4 (B) respectively; the COX defect is generalized but only weakly demonstrated histochemically. Scale bar = 50 *μ*m (C) Respiratory chain enzyme activity measurements in skeletal muscle from P1, P2, P3 and P4 demonstrate a combined enzyme defect involving complexes I and IV in all four patients compared to age‐matched controls. (D) Respiratory chain profile following quadruple oxidative phosphorylation immunofluorescence analysis of cryosectioned muscle from P4, confirming the presence of fibers lacking complex I (NDUFB8) protein, and to a lesser extent, complex IV (COXI) protein. Each dot represents the measurement from an individual muscle fiber, color‐coded according to its mitochondrial mass (blue‐low, normal‐beige, high‐orange, very high‐red). Dashed lines indicate SD limits for the classification of fibers. Lines next to x‐ and y‐axes represent the levels (SDs from the average of control fibers after normalization to porin/VDAC1 levels; _z= Z‐score, see Methods section of Rocha et al. 2015 for full description of statistics (https://www.ncbi.nlm.nih.gov/pubmed/26469001)) of NDUFB8 and COX1 respectively: (beige = normal (>−3), light beige = intermediate positive (−3 to−4.5), light purple = intermediate negative (−4.5 to −6), purple = deficient (<−6). Bold dotted lines indicate the mean expression level observed in respiratory‐normal muscle fibers. (E) Western blot of protein from fibroblasts showed reduced levels of complex I (CI) and complex IV (CIV) subunits in P5 relative to controls. Complex II subunit SDHB is indicative of loading. DT45 and LS1 were used as positive controls; DT45 has isolated complex I deficiency and mutations in *NDUFAF6*, and LS1 has isolated complex IV deficiency and mutations in *PET100*.

Twenty six of 28 patients (89%) with available muscle biopsies had evidence of respiratory chain deficiency (Fig.**** 2). Of these, complex I was decreased in all patients. Patterns of respiratory chain deficiency were as follows: isolated complex I deficiency (*n* = 9) and multiple respiratory chain deficiencies (*n* = 17). Complex I enzymatic activity was more severely decreased, compared to other complexes^8^ (Fig. 2C); this observation was corroborated with the findings of quadruple immunohistochemistry in one muscle biopsy where loss of complex I (NDUFB8) protein was more pronounced than loss of complex IV (COXI) protein (Fig. 2D). Three patients had muscle biopsies demonstrating no definite respiratory chain deficiency. Immunoblotting was performed in fibroblasts derived from 13 patients, identifying a quantitative loss of complex I and IV subunits in all cases (western blot of P5's fibroblasts is shown in Fig. 2E). Biochemical analysis of the OXPHOS enzymatic function in fibroblasts showed that the deficiency was more marked in complex I than complex IV for six of the seven cases tested.^6^, ^8^

### *MTFMT* pathogenic variants

**Figure 3:**
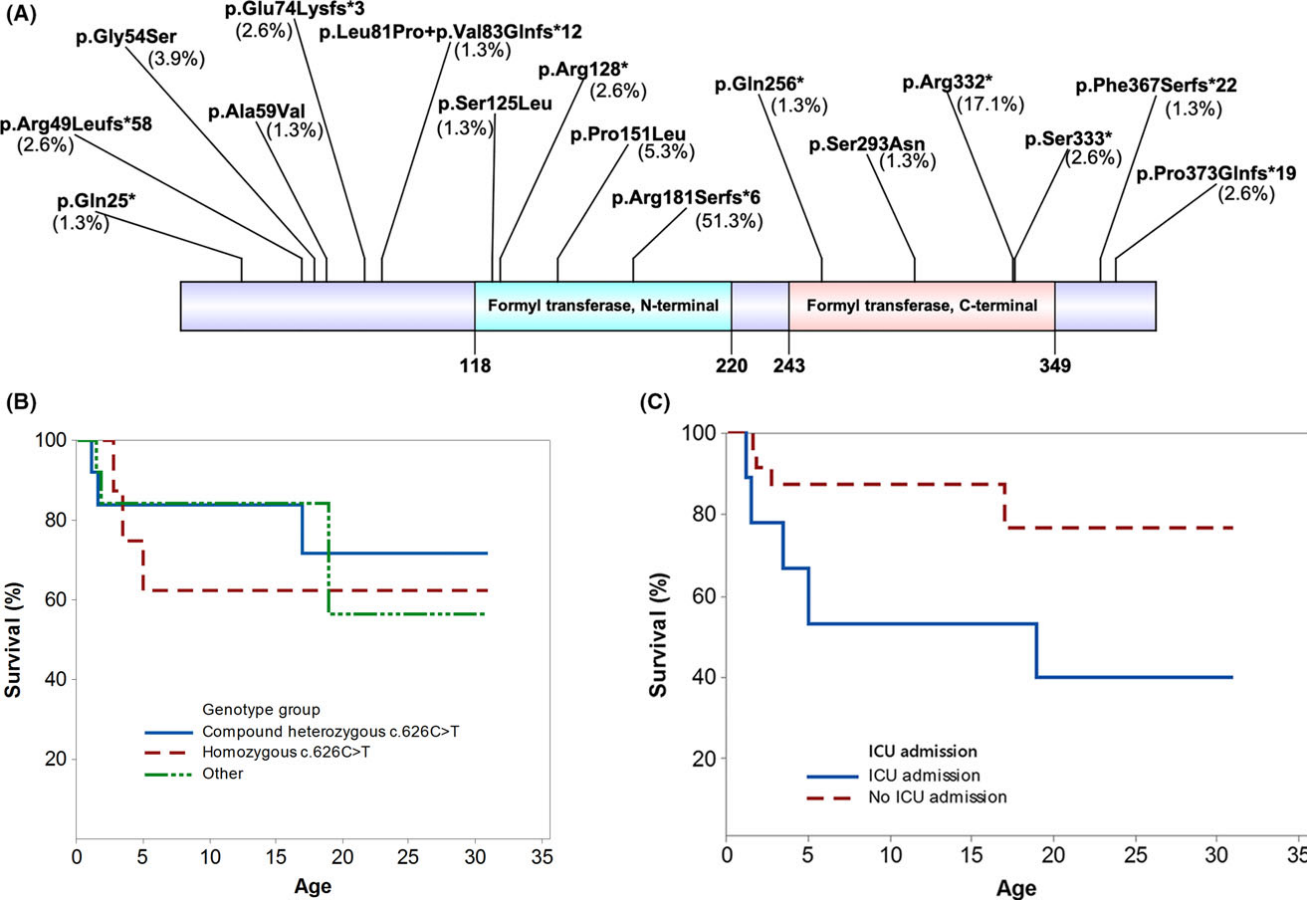
(A) Pathogenic variants in *MTFMT*. Sixteen pathogenic variants have been identified. The percentage in each bracket represents the frequency of a given pathogenic variant out of 76 alleles. Figure 3 (B) and (C) Survival Curves**.** Kaplan‐Meier curves show that no statistical difference in survival was observed in three different genotypic groups (B). Patients with a past history of ICU admission showed a nonsignificant trend towards shorter survival time when compared to those without ICU admission (*P* = 0.052, Log‐Rank) (C).

Of the 38 patients in this cohort, 30 were compound heterozygous and eight were homozygous for pathogenic *MTFMT* variants. There were 16 pathogenic variants: frameshift (*n* = 6), missense (*n* = 5) and nonsense (*n* = 5) (Fig. 3A). The c.626C>T is the most common variant, which has been identified in half of all cases. Homozygosity of c.626C>T was identified in seven patients; there was no report of consanguinity in any family with the exception of one patient in which the family history was not explicitly stated. The c.626C>T variant lies in exon 4, and predominantly results in the skipping of this exon by abolishing exonic splicing enhancers and/or by generating an exonic splicing suppressor.^6^ Skipping of exon 4 results in a frameshift and premature truncation of MTFMT (p.Arg181Serfs*6). A small amount of residual full‐length transcript is also produced where the c.626C>T variant encodes a protein with a p.Ser209Leu missense mutation.Figure 6 The explanation of amino acid nomenclature p.Arg181Serfs*6 is provided in the S1.

### Survival status

Twenty five of 34 patients (74%) were alive at the time of their last follow‐up (median 6.8 years, range: 14 months to 31 years, IQR: 14.5 years). The median age of death was 2.9 years (range: 14 months to 17 years, IQR: 9.45). The cause of death was known for five patients: generalized seizure (*n* = 1), pneumonia (*n* = 1), respiratory arrest (*n* = 1), cardiac arrhythmia (*n* = 1) and one patient died from an intracranial hemorrhage following a cardiac surgery. There was no statistical difference in survival status among three genetic subgroups: homozygous c.626C>T (*n* = 8), compound heterozygous c.626C>T (*n* = 13) and others (*n* = 13) (*P* = 0.744) (Fig. 3B). A history of ICU admission appeared to be associated with a poorer survival; however, this finding did not reach statistical significance (*P* = 0.052, Log‐Rank) (Fig. 3C). Other factors such as sex, seizures, imaging changes such as abnormalities of basal ganglia or brainstem, cardiac dysfunction, respiratory problems were not predictors of survival.

## Discussion

A recent multi‐center study in LS showed that the median age of onset was 7 months and nearly 40% of the patients died by the time of data analysis with median age of death being 2.4 years.^3^ In this retrospective, observational study, we demonstrate that patients with pathogenic *MTFMT* variants may have a later age of onset (median 14 months, range: birth to 17 years), with three patients presenting in their teenage years. Lactic acidosis, developmental delay, abnormal ocular findings and gait abnormalities were among the most common clinical findings. Abnormal cardiac investigations were identified in 53% of the patients and cardiomyopathy appeared to be the most common finding (35%). Furthermore, the survival rate appears higher in patients with *MTFMT*‐related mitochondrial disease with ~30% of the patients currently in their adulthood and ~25% died under the age of 21, compared to the previous cohort study.^3^ The rates of acute exacerbation and admission to ICU in our cohort were comparable to other case series of LS^3^; of the 24 patients who were documented to be alive, 14 of these had episodes of acute decompensation, and five had ICU admissions. This is particularly remarkable as LS is often associated with a rapid decline and poor prognosis, with death commonly occurring in preschool years.

Bilateral, symmetrical T_2_‐weighted hyperintensities of the basal ganglia and/or brainstem, are considered hallmarks of the syndrome and form part of the diagnostic criteria.^20^ Our findings show that 36% of the patients with *MTFMT* pathogenic variants did not have the typical basal ganglia signal abnormalities. Moreover, brainstem abnormalities were identified in only half of the patients. Around 20% of the patients had neither basal ganglia nor brainstem lesions noted. When present, MRI scans of the spine showed long, intrinsic signal abnormalities in the cervical and/or thoracic cord. However these were not routinely performed, only when patients presented with subacute spinal cord syndrome. They indicate that metabolic decompensation can also occur in spinal cord in addition to basal ganglia and brainstem, as shown in other mitochondrial genetic defects as well as postmortem spinal cord tissue from patients with LS.^21^, ^22^ White matter changes ranging from patchy changes to symmetrical, confluent subcortical white matter signal abnormality (leukodystrophy) were present in 55% of the patients presented here. The underlying neurobiological mechanism remains elusive as no neuropathological study is available to date. However, given MTFMT plays a part in one‐carbon metabolism, which requires folate as a key cofactor, it seems plausible that there might be a mechanistic link between MTFMT deficiency, oligodendrocyte dysfunction and impaired myelination.^23^

MTFMT deficiency has been previously proposed to be one of the common nuclear defects causing LS, and the c.626C>T variant was thought to be a founder genetic variant in the European population with a quoted allele frequency of 0.11%.^5^, ^8^ Interestingly, with the availability of larger exome and genome sequencing data (Genome Aggregation Database, gnomAD), the allele frequency for the c.626C>T in the non‐Finnish European population has not changed markedly (89/126686, 0.07%) and this variant has not been observed in the Ashkenazi Jewish, East Asian and South Asian populations. It is, therefore, unsurprising that at least six of the seven homozygous c.626C>T patients were from non‐consanguineous family pedigrees. Indeed, SNP array data pertaining to case 5 (see Data S2) demonstrate a small region of homozygosity (~90 kb) encompassing the c.626C>T variant (highlighted pink) without evidence of additional large stretches of homozygosity, consistent with a founder mutation opposed to undisclosed consanguinity. We have not identified any significant difference in survival in different pathogenic *MTFMT* variants, suggesting that genotype is not a predictor for the disease trajectory. Studies using a *Mtfmt* knockout mouse fibroblast model lacking exon 4 in *MTFMT* demonstrated that MTFMT is not an absolute requirement for initiation of translation and elongation of mitochondrial protein synthesis in mice but deficiency of this protein results in reduced efficiency of this process and downstream oxidative phosphorylation defects.^24^ This may contribute to the overall milder disease course observed in the *MTFMT*‐related mitochondrial disease presented here compared with typical LS.

Most of the patients in this case series had genomic studies performed after muscle biopsy had already provided evidence of a respiratory chain complex I or combined defect. However, current practice is increasingly to perform genomic studies earlier after clinical presentation in order to avoid an invasive biopsy where possible. For patients with *MTFMT* variants not previously linked to disease it may be necessary to obtain functional evidence to prove causality. Muscle biopsy can still be avoided as immunochemical analysis with a respiratory chain antibody cocktail demonstrated loss of complex I and IV subunits in fibroblasts from all 13 patients in this series studied in this way. That profile is shared by many other defects of mitochondrial translation so it is worth noting that almost all *MTFMT* patients reported to date have had mutations that are expected to result in substantial loss of MTFMT protein. Haack and colleagues showed that fibroblasts from all five patients tested had markedly decreased MTFMT on western blot, providing a specific test for loss of function variants.^8^

We acknowledge the limitations of our study, including the retrospective design and the variable follow‐up of patients resulting in incomplete clinical or biochemical data for some cases. This, however, suggests that the frequency of clinical symptoms described here is likely to be an underestimation. Despite our best effort of enrolling new patients and previously reported cases in the regression analysis, we have not been able to identify any significant predictors for the survival.

In conclusion, our findings demonstrate that patients with pathogenic variants in *MTFMT* present at a relatively older age and exhibit a milder disease trajectory with 29% of the patients surviving into adulthood, compared to other nuclear genetic defects that cause LS. Eighteen percent of the patients exhibited a classical clinical course without typical radiological findings of LS, highlighting that this condition may still be under‐recognized. Fibroblasts may preclude the need for muscle biopsy, when novel variants are identified, to confirm the pathogenicity.

## Strobe Statement

STROBE guidelines were adhered to in the write‐up and analysis of this observational, cohort study

## Patient Consent

Obtained.

## Authors Contributions

Conception or design of the work: GSG, RM, RWT and YSN; data acquisition, analysis and interpretation: all authors; drafting the manuscript: HH, DRT, GSG, RM, RWT and YSN; critical review and final approval of the manuscript: all authors; RWT and YSN are listed as guarantors of the paper.

## Conflict of Interest

None declared.

## Supporting information


**Figure S1.** Explanation for the change in *MTFMT* mutation nomenclature.

**Table S1.** Summary of eight new cases (P1,2,5,6,7,8,10,11) and 30 other previously reported patients.

**Data S1.** Clinical vignettes (Patients 1‐14)

**Data S2.** SNP array data demonstrate a small region of homozygosity (~90kb) encompassing the c.626C>T variant (highlighted pink) without evidence of additional large stretches of homozygosity.
