# Supplementary material for: Leigh syndrome caused by mutations in MTFMT is associated with a better prognosis
Source: Ann Clin Transl Neurol. 2019 Feb 17;6(3):515–24. doi: 10.1002/acn3.725 (PMC6414492; doi:10.1002/acn3.725)
Supplement: Supplementary file 1 — Figure S1. Explanation for the change in MTFMT mutation nomenclature. [file ACN3-6-515-s001.pdf]

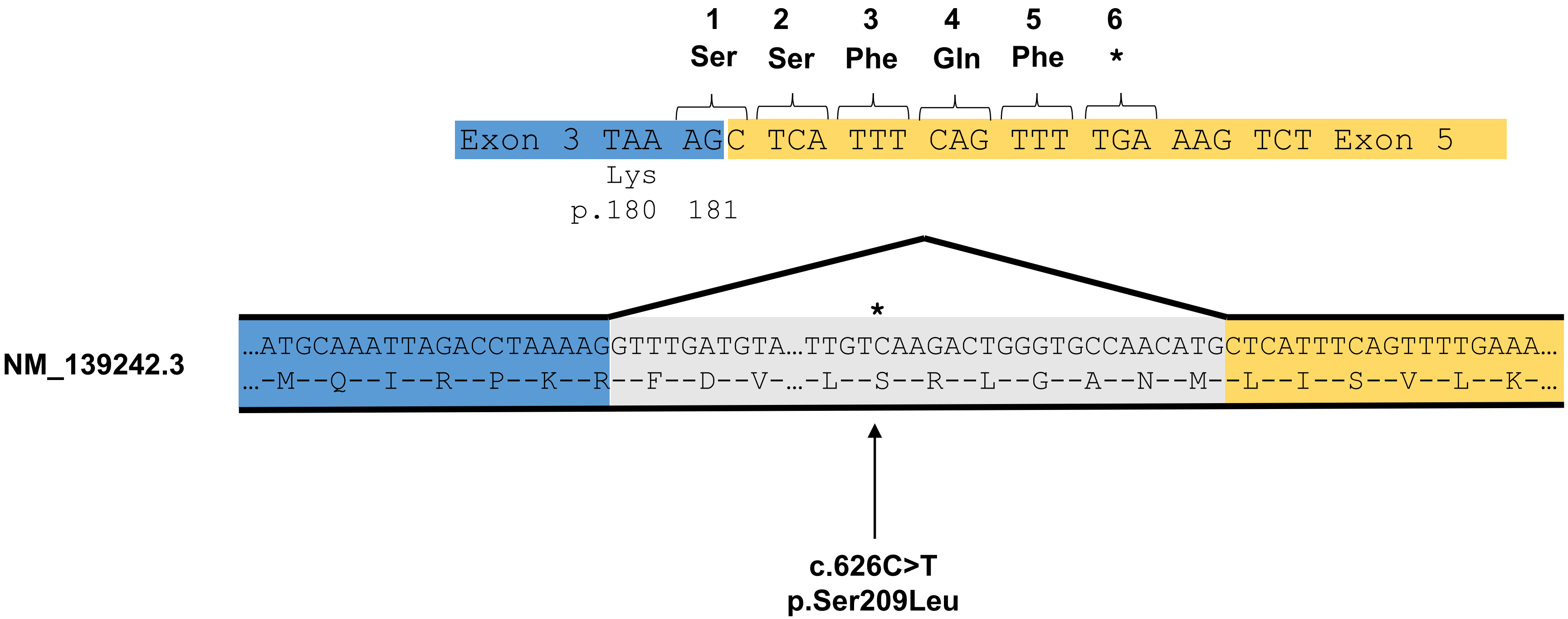

Supplementary Figure 1: Explanation for the change in *MTFMT* mutation nomenclature.
The common c.626C>T substitution results in two mRNA transcripts, one that harbours a p.Ser209Leu missense variant (lower heavy line), and another exhibiting skipping of exon 4 (upper heavy line). Skipping of exon 4 causes a frameshift at p.181, changing the p.181 codon AGG (Arg) to AGC (Ser), p.Arg181Ser; the frameshift covers a further four residues before a stop codon is introduced. Six residues are affected, hence p.Arg181Serfs*6.
Exon 3, shaded blue; Exon 4, shaded grey, Exon 5 shaded yellow.
