# Supplementary material for: Leigh syndrome caused by mutations in MTFMT is associated with a better prognosis
Source: Ann Clin Transl Neurol. 2019 Feb 17;6(3):515–24. doi: 10.1002/acn3.725 (PMC6414492; doi:10.1002/acn3.725)
Supplement: Supplementary file 2 — Table S1. Summary of eight new cases (P1,2,5,6,7,8,10,11) and 30 other previously reported patients. [file ACN3-6-515-s002.docx]

| Institute/ Paper | Patient ID | *MTFMT* pathogenic variant | | Age of onset | Age at last follow up | Presenting symptoms | Mobility | LA | Cardiac abnormality | Muscle biopsy |
| --- | --- | --- | --- | --- | --- | --- | --- | --- | --- | --- |
|  |  | **Nucleotide** | **Amino Acid** |  |  |  |  |  |  |  |
| UK | 1^^, C^ | c.626C>T  c.626C>T | p.Arg181Serfs*6  p.Arg181Serfs*6 | 9m | 3y6m^+^ | IUGR, DD, hypotonia | Deceased | Yes | Cardiomyopathy | CI, CIV |
|  | 2^^, C^ | c.626C>T  c.626C>T | p.Arg181Serfs*6  p.Arg181Serfs*6 | 3y | 6y | DD | Independent | Yes | Normal | CI, CIV |
|  | 3 (P30/ P19)^A, C^ | c.626C>T  c.1100_1101delTT | p.Arg181Serfs*6  p.Phe367Serfs*22 | 9m | 26y | DD | Independent | Yes | Cardiomyopathy, sinus tachycardia | CI, CIV |
|  | 4^A, C^ | c.626C>T  c.626C>T | p.Arg181Serfs*6  p.Arg181Serfs*6 | 2y | 8y | DD | Crawling where possible | Yes | Normal | CI, CIV |
| Australia | 5^^, C^ | c.626C>T  c.626C>T | p.Arg181Serfs*6 p.Arg181Serfs*6 | 6m | 2y9m^+^ | DD | Deceased | Yes | Cardiomyopathy | CI |
| Poland | 6^^, C^ | c.994C>T  c.994C>T | p.Arg332*  p.Arg332* | 9m | 22m^+^ | DD, hypotonia, dysmorphic features | Deceased | Yes | Not known | CI, CIV |
|  | 7^^, C^ | c.626C>T  c.994C>T | p.Arg181Serfs*6  p.Arg332* | 15m | 2y4m | DD, hypotonia | Loss of ambulation | Yes | Not known | N/A |
|  | 8^^, C^ | c.626C>T  c.994C>T | p.Arg181Serfs*6  p.Arg332* | 18m | 4y | DD, apathy, convergent strabismus | Loss of ambulation | Yes | Cardiomyopathy | CI, CIII, CIV |
|  | 9^A, C^ | c.626C>T  c.994C>T | p.Arg181Serfs*6  p.Arg332* | 6m | 18m^+^ | Hypotonia, developmental regression | Deceased | Yes | Cardiomyopathy, WPW | Low CS activity |
| USA | 10^^, C^ | c.626C>T  c.998G>C | p.Arg181Serfs*6 p.Ser333* | 2w | 8y | Feeding difficulties, dysmorphic features, DD | Independent | Yes | Bradycardia | N/A |
| Netherlands | 11^ | c.626C>T  c.994C>T | p.Arg181Serfs*6  p.Arg332* | Birth | 31y | VSD | Independent | Yes | VSD | N/A |
|  | 12^A^ | c.160G>A  c.626C>T | p.Gly54Ser  p.Arg181Serfs*6 | 3y | 16y | Autistic spectrum disorder | Independent | Yes | No | Increased CS |
|  | 13^A^ | c.160G>A  c.626C>T | p.Gly54Ser  p.Arg181Serfs*6 | 4y | 19y^+^ | Autistic spectrum disorder | Deceased | Yes | SVT | CI |
|  | 14^A^ | c.160G>A  c.626C>T | p.Gly54Ser  p.Arg181Serfs*6 | 7y | 25y | Exercise intolerance | Independent | Yes | No | CI, CIII |
| Tucker (2011) | 15^A^ | c.374C>T  c.626C>T | p.Ser125Leu p.Arg181Serfs*6 | 5y | 5y^+^ | Weight gain, hypertension | Deceased | Yes | Not known | CI, CIII, CIV |
|  | 16^A^ | c.626C>T  c.382C>T | p.Arg181Serfs*6 p.Arg128* | 9y | 21y | Strabismus, decreased VA, DD | Independent | Yes | WPW | CI, CIII |
|  | 17^A^ | c.626C>T  c.382C>T | p.Arg181Serfs*6 p.Arg128* | 9y | 18y | DD, optic atrophy | Independent | No | WPW | CI, CIV |
| La Piana (2017) | 18 | c.626C>T  c.176C>T | p.Arg181Serfs*6 p.Ala59Val | 7y | 24y | DD, cranial nerve IV palsy | Not known | Not known | Not known | CI, CIV |
| Oates (2016) | 19 | c.1116delT  c.1116delT | p.Pro373Glnfs*19 p.Pro373Glnfs*19 | Birth | 4y | Oligohydramnios | Not known | Not known | Pulmonary stenosis | Not known |
| Pena (2016) | 20 | c.626C>T  c.626C>T | p.Arg181Serfs*6  p.Arg181Serfs*6 | 2y | 19y | Nystagmus | Maximal assistance | Yes | Not known | Normal |
| Hinttala (2015) | 21 (I) | c.626C>T  c.994C>T | p.Arg181Serfs*6  p.Arg332* | N/A | N/A | N/A | N/A | N/A | N/A | N/A |
|  | 22 (II) | c.242T>C+c.247_253del    c.994C>T | p.Leu81Pro+ p.Val83Glnfs*12  p.Arg332* | N/A | N/A | N/A | N/A | N/A | N/A | N/A |
|  | 23 (III) | c.219_222del  c.626C>T | p.Glu74Lysfs*3 p.Arg181Serfs*6 | N/A | N/A | N/A | N/A | N/A | N/A | N/A |
| Haack (2014) | 24 (73922) | c.452C>T  c.626C>T | p.Pro151Leu  p.Arg181Serfs*6 | Birth | 15m | Hypotonia | Not known | Yes | Cardiomyopathy | CI, CIV |
|  | 25 (8432723) | c.626C>T  c.766C>T | p.Arg181Serfs*6  p.Gln256* | Birth | 12y | Apnoea, bradycardia, fatigue | Broad based gait | Yes | Bradycardia | CI |
|  | 26 (49728) | c.626C>T  c.878G>A | p.Arg181Serfs*6  p.Ser293Asn | 2m | 14m^+^ | Lack of fixation, microcephaly | Deceased | Yes | Septal thickening | CI, CIV |
|  | 27 (52181) | c.219_222del  c.626C>T | p.Glu74Lysfs*3  p.Arg181Serfs*6 | 2m | 19m^+^ | DD | Deceased | Yes | Not known | CI |
|  | 28 (54502) | c.626C>T  c.994C>T | p.Arg181Serfs*6  p.Arg332* | 3m | 6y | DD, hypotonia | Not known | Yes | Cardiomyopathy | CI, CIV |
|  | 29 (56713) | c.73C>T  c.626C>T | p.Gln25*  p.Arg181Serfs*6 | 8m | 6y | Hypotonia, spasticity, DD | Broad based gait | Not known | No | CI |
|  | 30 (61606) | c.626C>T  c.626C>T | p.Arg181Serfs*6  p.Arg181Serfs*6 | 16m | 6.5y | DD, Abnormal gait | Not known | Not known | Not known | CI |
|  | 31 (56902) | c.452C>T  c.994C>T | p.Pro151Leu  p.Arg332* | 20m | 5y | Gait instability during infection | Not known | Yes | Cardiomyopathy | CI, CIV |
|  | 32 (44409) | c.626C>T  c.994C>T | p.Arg181Serfs*6  p.Arg332* | 3y | 24y | DD | Not known | Yes | Not known | CI |
|  | 33 (33467)^S^ | c.146_153del  c.626C>T | p.Arg49Leufs*58  p.Arg181Serfs*6 | 15y | 22y | Unilateral squint | Not known | Yes | Not known | CI |
|  | 34 (33009)^S^ | c.146_153del  c.626C>T | p.Arg49Leufs*58  p.Arg181Serfs*6 | 17y | 17y^+^ | DD, visual disturbance, fatigue | Deceased | No | Aortic regurgitation, VSD | CI |
| Prasun (2014) | 35 | c.626C>T  c.626C>T | p.Arg181Serfs*6  p.Arg181Serfs*6 | 17y | 17y | Blurred vision, dysphagia | Not known | Not known | Tachycardia | CI, CIII, CIV, CV |
| Neeve (2013) | 36 (I)^S^ | c.452C>T  c.994C>T | p.Pro151Leu  p.Arg332* | 3y | 16y | DD | Ataxic gait | Yes | No | CI, CIV |
|  | 37  (II)^S^ | c.452C>T  c.994C>T | p.Pro151Leu  p.Arg332* | 5y | 6y | DD | Independent | Yes | Not known | Not known |
| DaRe (2013) | 38 | c.626C>T  c.998G>C | p.Arg181Serfs*6 p.Ser333* | N/A | N/A | N/A | N/A | N/A | N/A | Not known |

**Supplemental Table 1: Summary of eight new cases (P1,2,5,6,7,8,10,11) and 30 other previously reported patients.** ^=new case, ^+^=deceased, A= additional information, C= clinical summary available in supplementary material, CS= citrate synthase, DD= developmental delay, IUGR= intrauterine growth restriction, LA= lactic acidosis, LS= Leigh syndrome, LV = left ventricle, N/A = not available, S=siblings, SVT= supraventricular tachycardia, WPW = Wolff-Parkinson-White syndrome, VA= visual acuity, VSD= ventricular septal defect
