# Supplementary material for: Leigh syndrome caused by mutations in MTFMT is associated with a better prognosis
Source: Ann Clin Transl Neurol. 2019 Feb 17;6(3):515–24. doi: 10.1002/acn3.725 (PMC6414492; doi:10.1002/acn3.725)
Supplement: Supplementary file 3 — Data S1. Clinical vignettes (Patients 1‐14) [file ACN3-6-515-s003.docx]

**Supplemental Material: Patient Case Summaries**

Patient 1

Patient 1 was born pre-term at 32+3 weeks. The pregnancy was complicated by intrauterine growth restriction, and he required non-invasive ventilation for three weeks. He failed the newborn hearing assessment and was noted to have hypospadias. He presented with delayed motor milestones followed by regression of skills and hypotonia at 9 months. Blood and CSF lactate were 4 and 6 mmol/L respectively, and MRI revealed abnormalities in the white matter. At age 18 months it was noted that his skills had been rapidly declining over the previous months.

He showed significant improvement at age 2 years, in terms of regaining skills previously lost as well as acquiring new skills. He was able to say a small number of words and pass from one hand to the other, although did show a preference for his right hand. He had a gastrostomy inserted due to poor feeding and was completely nil by mouth. Clinical examination revealed profound central hypotonia with an increase in tone in all four limbs. There was no evidence of dystonia or hyperkinetic movements. He underwent a muscle biopsy that showed multiple respiratory chain deficiencies (complexes I and IV), and exome sequencing revealed homozygous mutations in *MTFMT* gene (c.626C>T).

He developed seizures that were precipitated by infection at age 3 years. He was intubated and ventilated in the intensive care unit for three days. There were concerns regarding abnormal breathing patterns at this time, and a sleep study suggested there were some desaturations. He was hypertensive and an echocardiogram identified left ventricular hypertrophy. It was felt that he regressed developmentally following this admission, and also developed a unilateral squint. He died age 3 years and 6 months.

Patient 2

Patient 2 was born at 41 weeks plus 2 days gestation. The pregnancy was complicated by a breech delivery and she was admitted to the special care baby unit initially. Her birth weight was 5lb 4oz (2.38kg). She had met the initial developmental milestones but only managed to walk independently until 20 months. She presented age 3 years with motor developmental delay; in particular she was unsteady and had frequent falls. Her MRI at this time showed symmetrical T2-hyperintenities in the lentiform nuclei, asymmetrical changes in the caudate nuclei and patchy deep white matter changes. Cerebospinal fluid (CSF) examination showed raised lactate at 4.3mmol/L (normal <2.2. mmol/L). Mitochondrial disease was suspected and a muscle biopsy was performed, which showed combined deficiencies of mitochondrial complexes I and IV.

At age 4, she walked with an in toeing gait and was noted to have a dystonic posture of her fingers when her arms were outstretched. She was unsteady and had bilateral dysdiadochokinesis. Her reflexes and eye movements were normal. Cardiology and ophthalmology review revealed no abnormality. She was initially commenced on Biotin and thiamine but these were discontinued and she remains on ubiquinone 30mg TDS.

Exome sequencing identified homozygous pathogenic variants in *MTFMT*. By the age of 5 years she made good developmental progress; although her gait remained immature and she could not jump of hop, her running appeared more coordinated than previous. On last review at the age of 6 years her gait was normal although eye closure provoked some unsteadiness. She had no speech or cognitive problems, tone and reflexes were all within the normal limits although she had slight proximal weakness particularly on rising from a squat.

Patient 3

Patient 3 was born at term; she was noted to be jaundiced 24 hours after birth, and parents noted she would tire significantly after feeding and vomit. She did not reach the expected developmental milestones at 9 and 12 months. In addition, she did not speak until 5 years old. It also became evident that she had moderate learning difficulties and a short attention span. CT head performed at age 5 years indicated possible brain damage. A repeat scan revealed basal ganglia calcification and changes in the cerebral peduncles three years later. Both the CSF and serum lactate were elevated, and muscle biopsy showed complex I on the low end of normal range. She developed precocious puberty and was given Goserelin (luteinizing hormone releasing hormone agonist) until age 13 years.

At age 17 years, she developed oculogyric episodes over a few months, which a frequency of few times a day. These involved in-turning of the right eye, and, on examination it was noted that she had limited upgaze and marginally limited abduction in the right eye. This fluctuated in severity but became worse later in the day. Fundoscopy examination was normal. At this age, she also developed urinary retention requiring intermittent catheterisation, which resolved 6 months later. Other new symptoms included headache, tremor, bilateral myoclonic jerks and pain in lower limbs.

She was tachycardic at a rate of 111 beats per minute and experienced palpitations and episodes of chest pain responsible for three hospital admissions at the age of 20 years. Bisoprolol was initially given to treat the tachycardia and palpitations after the first consultation. After this an echocardiogram was performed which showed a regional wall abnormality, indicative of secondary cardiomyopathy. The dose of bisoprolol was increased and it was eventually replaced by ivabradine.

A repeat muscle biopsy showed a marked respiratory chain defect in complexes I and IV. Sequential COX/SDH histology demonstrated 60% of fibres with COX intermediate or transitions. An MRI scan was done in January 2012, which showed Leigh’s like changes: bilateral necrotic changes within the putamen and periaqueductal region midbrain changes. Additionally there was increased signal in the cervical spinal cord. An EEG revealed excessive polyrhythmic appearances with no evidence of epileptiform activity. NCS showed mild to moderate sensory neuropathy (predominantly in the lower limbs) for which gabapentin was prescribed to treat the pain. EMG studies revealed mild myopathy.

As part of the diagnostic workup for investigating the signal abnormality in the cervical cord, a lumbar puncture was performed and it showed an elevated opening pressure (58cm H_2_O) and normal CSF constituents. A diagnosis of idiopathic intracranial hypertension was made after excluding other causes. She was initially managed with medical therapy, followed by the lumbar-peritoneal shunt insertion.

Exome sequencing identified compound heterozygous pathogenic variants in *MTFMT* (c.626C>T and a novel 2bp deletion (c.1100_1101)). Her disease progressed slowly with slight worsening of myoclonic jerks, and she developed bilateral tremor and ataxia. A DAT scan was performed and the reduction of dopamine uptake was detected in the basal ganglia. Repeated nerve conduction studies showed sensory axonal neuropathy and dorsal root ganglionopathy. She was last reviewed age 26 years and was walking independently.

Patient 4

Patient 4 was born at 40+6 weeks by caesarean section following a normal pregnancy but failure to progress during labour. He had motor and speech developmental delay; he only started to walk at 22 month, and the circumduction gait was observed. At age 2, he was referred to the paediatric service for the review of his gait. His left foot was splinted. However, at age 3 years he was noted to have dystonia affecting his arms, and increased tone in both legs. Blood and CSF lactate were elevated and muscle biopsy showed complex I and IV deficiencies. MRI at this time showed bilateral T2-hyperintensities in putamen and caudate nuclei, with a restricted diffusion. In addition, there were patchy white matter changes identified in the genu of corpus callosum and centrum semiovale.

At age 4 years he had a sudden deterioration with an increase in falls. He was noted to be walking on his toes and to have a tremor. He has since had splints applied to both feet and this seems to have improved his balance and gait. On review age 4 years 10 months he was unable to run or jump. He could mark with a pen but unable to copy shapes, and had a tripod grip. He could put 4 or 5 words together in a short sentence. Hearing and vision were normal. He suffered from sleep disturbance with waking frequently, but was able to attend a mainstream school with one to one support. On examination he had bilateral increased tone in his legs but reflexes were intact. He was commenced on a trial of ubiquinone that appeared to increase his physical activities.

He showed some improvement in speech and communication when he was reviewed at age 5 years. Clinical examination showed brisk reflexes and extensor plantar responses. He had dystonic posturing and walked with his left heel slightly elevated and left arm flexed at the elbow. Genetic testing revealed homozygous mutations in *MTFMT*. He was commenced on baclofen and trihexyphenidyl which somewhat improved the dystonia.

His mobility deteriorated and had frequent falls at age 6 years. He preferred to crawl than walk. Gait was more dystonic and feeding slowed. He had significant spasticity of both legs on examination. He was stable when last reviewed age 8 years.

Patient 5

Patient 5 was the third child of unrelated parents and was born at term following an uncomplicated pregnancy. Developmental delay was noticed at 6 months when he had not reached appropriate milestones. At 12 months of age he was able to sit but not roll, at 18 months he was able to stand, and at 32 months he developed an ataxic wide-based gait. He was able to speak 6 to 10 understandable words by 33 months of age.

He developed recurrent respiratory problems including a number of transient episodes of pallor and cyanosis with spontaneous recovery. At 2 years 9 months he presented with a prolonged hypoxic episode with cyanosis, hypotonia, and loss of consciousness. Due to central hypoventilation, he was admitted to the ICU where he remained ventilator dependent. On examination at that time, he was hypotonic but was moving all limbs, a right divergent squint was noted and fundus evaluation showed bilateral optic atrophy. There was no upper airway lesion on direct laryngoscopy. There was a grade 2/4 systolic murmur at the upper left sternal edge. Peripheral reflexes were present and symmetrical.

A CT scan of his head demonstrated areas of poor attenuation in the midbrain region raising the possibility of mitochondrial disease. He had a CSF lactate of 4.0 mmol/L (reference range <1.7), and a CSF lactate/pyruvate ratio of 33.3. ECG and echocardiography showed biventricular hypertrophy with no congenital structural abnormalities. Further investigations included normal plasma lactate, liver function tests, creatinine kinase, ammonia, thyroid function testing, urine metabolic screening, amino acids, white cell lysosomal enzymes, and karyotype.

During this hospital admission he had a right sided tonic-clonic seizure that ceased with diazepam. Multiple attempts to wean the patient off ventilation failed and his spontaneous breathing rate was 1 -3 breaths/minute. He was unable to swallow his saliva. In view of the poor prognosis, he was transferred to the family home where he was extubated and died a short time after.

Patient 6

Patient 6, the second child of healthy unrelated parents, was born at term following emergency caesarean section for maternal haemolysis, elevated liver enzymes and low platelets (HELLP syndrome). He was healthy at birth, although was noted to have dysmorphic features including low-set ears, narrow lips, finger nail atrophy, foot syndactyly, hypospadias and cryptorchidism. Psychomotor development was delayed from infancy, with developmental regression, muscular hypotonia, periodic nystagmus and tachypnea observed from 9 months of age. MRI head showed symmetrical high signal on T2 lesions supraventricular in the white matter, with similar changes in the pons and medial parts of the temporal lobes. Plasma lactate was elevated. EEG was normal. Muscle biopsy showed complex I and IV deficiency.

He continued to deteriorate, and at the age of 10 months was fed with nasogastric tube due to swallowing difficulties and weight loss, and then went on to have a percutaneous endoscopic gastrostomy (PEG) inserted. He had no episodes of acute metabolic decompensation but progression of the disease included more frequent vomiting and respiratory infections pneumonia. He was treated with vitamins D, A and E, thiamine and coenzyme Q10. A ketogenic diet was attempted but discontinued due to poor tolerance. He died age 22 months, and the molecular diagnosis of a mutation in *MTFMT* was established post-mortem by exome sequencing.

Patient 7

Patient 7 was born by planned caesarean section at term. He was noted as a neonate to have right eyelid drooping, but development was otherwise normal for the first year.

At the age of 15 months he was noted to have a left-sided spastic paresis and torticollis. After respiratory and ear infections he stopped crawling and sitting independently, and was found to have muscular hypotonia and pyramidal symptoms. MRI head showed symmetrical high signal lesions on T2 in the supraventricular white matter. Similar changes were also seen in the corupus callosum, caudate, putamen and globus pallidus and crus cerebri. Serum and CSF lactate was elevated.

His condition deteriorated and he required ICU admission with intubation and tracheostomy formation at the age of 2 years and 4 months due to progressive respiratory failure in the context of an infection. On last review he was being managed at home with respiratory support and PEG feeding, and repeat MRI showed new lesions in the spinal cord.

Patient 8

Patient 8 was the first child of healthy unrelated parents and was born at term. At 1 month old he was diagnosed with hypertrophic cardiomyopathy. Developmental milestones were reached initially, however at the age of 18 months his parents observed speech and language delay, apathy and drowsiness. He was noted to be less active during the day and made poor eye contact. On neurology review aged 20 months he was found to have hypotonia, convergent strabismus and difficulties in establishing eye contact. EEG was normal. MRI examination revealed symmetrical high signal on T2 lesions involving the lentiform nuclei, caudate nuclei, mid-brain and crus cerebri. Plasma lactate was elevated. Muscle biopsy revealed deficiencies in complexes I, III and IV.

Gradual regression in the child's development was observed: he stopped walking alone at the age of 21 months then had difficulties in sitting independently, with further regression of speech and swallowing. At the age of 3 years, in the context of an acute infection, he developed respiratory failure requiring intensive care admission. At the age of 4 years he has a tracheostomy and is PEG fed.

Patient 9

Patient 9 was born at term, the first child of healthy unrelated parents. Neonatal period was uneventful. At 2 months he was being treated for pneumonia and was found to have cardiomyopathy. At this time development was normal. At the age of 6 months, after an infection, he was noted to have developmental regression, and a CT head scan showed changes in white matter. Pigmentary degeneration of retina was also identified. Plasma and CSF lactate were elevated. Muscle biopsy showed small hypotrophic muscle fibers with increased fatty and fibrous tissues.

At the age of 15 months he was hospitalised due to dyspnoea in the context of pneumonia. Antibiotic therapy was commenced, initially improving the clinical condition, however on the 5^th^ day of his hospital admission there was a sudden deterioration of his condition, with gastrointestinal bleeding and gastrointestinal obstruction. Due to the accumulation of metabolic and respiratory acidosis and deteriorating condition he was transferred to the ICU, where he was found to have renal impairment, heart failure and diabetes. The boy died at the age of 18 months due to multiple organ failure. The molecular diagnosis of *MTFMT* was established post mortem by WES.

Patient 10

This patient was born at term via caesarean section due to concerns about intrauterine growth restriction (IUGR). His mother had previously had six first trimester miscarriages attributed to a pro-thombotic state.

He had facial dysmorphia and hypospadias noted at birth. At two weeks he presented with feeding difficulties and apnoeic episodes, likely due to laryngomalacia, and he was found to have a hiatus hernia treated surgically with fundoplication. He had a tracheostomy at 2 months of age. He had episodes of extension and tensing starting at one month old, and was diagnosed with infantile spasms at 3 months. He was commenced on Oxcarbazepine aged 18 months.

Global developmental delay was noted in infancy; this fluctuated and at times he lost skills but would later regain some of these. He could say 20-25 words at age 2.5 years but currently only has 4 words. He was cruising at 3 years old, and taking independent steps at 3.5 years, although periodically would become unsteady.

Seizures have remained well controlled although he has had some episodes of “jerking” with a normal EEG. Tracheostomy was removed at 2.5 years. He is currently fed via an NG tube and has episodes both of constipation and diarrhoea. More recently, the mother has been concerned about episodes of apnoea although he has had two normal sleep studies.

Patient 11

Patient 11 was born of unrelated healthy parents. Ventricular septal defect and diaphragmatic hernia were identified at birth. Her other significant past medical history included failure to thrive, short stature, and mild intellectual disability. At the age of 27 years, she presented with a gradual-onset visual disturbance that evolved over a few weeks. On examination, she had ophthalmoplegia, a diminished facial expression, bradykinesia and rigidity in both arms. Her MRI head showed mesencephalic tegmental T2-hyperintense lesions, which were initially thought to be thiamine deficiency and replacement therapy was started. Whilst there were some resolutions of her symptoms after the thiamine replacement, a follow‐up MRI head demonstrated one month later identified more extensive imaging abnormalities a month later. There were hyperintensities in the mesencephalon, thalamus, substantia nigra, colliculus inferior and colliculus superior. She had elevated CSF lactate (4.2 mmol/l, normal range < 2 mmol/L), elevated alanine level in both CSF (63.3 μmol/l, normal: 19.54‐53.75) and serum (812 μmol/l, normal range: 150‐450), and elevated malate acid in urine (31 μmol/mmol creat, normal range: 0‐15). A mitochondrial encephalopathy was suspected.

Four months after the initial presentation, she was admitted to hospital with pneumonia and became respiratory distress necessitating mechanical ventilation. She developed dysphagia, bilateral facial weakness and quadriplegia. She was treated with Ceftriaxone intravenously and the pneumonic changes on the chest radiograph improved. However, her neurological symptoms and signs persisted. She had marked ptosis bilaterally and almost complete facial paralysis. Weaning off the mechanical ventilation was unsuccessful due to severe dysphagia and poor coughing reflex. However, the patient was fully conscious and able to answer questions by nodding the head. Subsequent brain imaging showed a progression of the hyperintense lesions in the mesencephalon and the medulla compared to the previous scans. She was empirically treated with thiamine, vitamin B complex, biotin and riboflavin, but there was no evidence of clinical response.

However, she showed remarkable recovery three months after the hospital admission. Her ophthalmoplegia and facial weakness resolved almost completely, her limbs were much stronger, and she was able to walk with some support, but still required ventilatory support. The resolution of MRI abnormalities mirrored the clinical improvement. She was referred for home‐artificial breathing, and only require nocturnal non-invasive ventilation six months later. Whole exome sequencing was performed and identified compound heterozygous mutations in *MTFMT*.

Patient 12

Patient 12 was born uneventfully, and he is the youngest child in the family. He was suspected to have the same disease as his sibling (Patient 14, proband) at age three years. He exhibited autism-like symptoms with a mild intellectual disability and an anxiety disorder. He had increased tone in his limbs with brisk reflexes and bilateral extensor plantar response. There was no evidence of dystonia. His MRI head demonstrated discrete T2 hyperintensities of the putamina.

His disease course is stable. He needs psychiatric support for schizophrenia.

Patient 13

Patient 13 was born at term and is the brother of Patients 12 and 14. He has been diagnosed with autism spectrum disorder as part of the complex developmental disorder. At primary school, he developed psychosis. On clinical examination, he had brisk bilateral reflexes with monotonic voice, lack of facial expression and generalised rigidity. He harboured the same *MTFMT* mutations as his other siblings.

He deteriorated rapidly following a viral infection; he developed an abnormal breathing pattern and cardiac arrhythmia (premature ventricular complexes and supraventricular tachycardia). He died at the age of 19 from cardiorespiratory failure.

Patient 14

Patient 14 was born at 34 weeks as the second child of unrelated parents. Patient 13 and 14 are his younger brothers. A difficulty of sucking was documented after birth. At the age of four years, he was admitted to hospital with severe sepsis secondary to pneumonia. He was found to have global developmental delay. At ten years old, he was documented to have fatigue, intellectual disability, bradyphrenia, hypokinetic with dystonic posturing, and unilateral resting tremor. He also exhibited an abnormal breathing pattern with frequent sighing as well as having speech and swallowing difficulties. He received carnitine, Vitamin E, A, folic acid and co-enzyme Q10. The symptoms of Parkinsonism showed reasonable response with levodopa. He had a CT head which showed hypointensities in the putamen and globus pallidus. His serum lactate was mildly elevated at 2.5mmol/L (normal < 2 mmol/L). His clinical phenotype was compatible with Leigh syndrome, and his muscle biopsy demonstrated complex I and III deficiencies. Whole mitochondrial DNA genome sequencing excluded pathogenic mutations and whole exome sequencing identified compound heterozygous mutations in the *MTFMT* gene. He required gastrostomy feeding because of the dysphagia. He became wheel-chair dependent as his Parkinsonism advanced. His MRI head performed at the age of 16 years only showed T2-hyperintensity in the periaqueductal area.
